# Metabolomics Analysis Coupled with Weighted Gene Co-Expression Network Analysis Unravels the Associations of Tricarboxylic Acid Cycle-Intermediates with Edible Pigments Produced by *Monascus purpureus* (Hong Qu)

**DOI:** 10.3390/foods11142168

**Published:** 2022-07-21

**Authors:** Hao Zhang, Huanhuan Liu, Lin Shu, Huimin Xu, Ying Cheng, Zhitao Mao, Bin Liu, Xiaoping Liao, Di Huang

**Affiliations:** 1 Department of Physical Education, Shenyang Ligong University, Shenyang 110159, China; zhanghao788109@163.com; 2 State Key Laboratory of Food Nutrition and Safety, Tianjin University of Science & Technology, Tianjin 300457, China; lh_tust@tust.edu.cn (H.L.); shulin@mail.tust.edu.cn (L.S.); xuhuimin@mail.tust.edu.cn (H.X.); 20844965@mail.tust.edu.cn (Y.C.); 3 Key Laboratory of Systems Microbial Biotechnology, Biodesign Center, Tianjin Institute of Industrial Biotechnology, Chinese Academy of Sciences, Tianjin 300308, China; mao_zt@tib.cas.cn; 4 TEDA School of Biological Sciences and Biotechnology, Nankai University, TEDA, Tianjin 300457, China; liubin1981@nankai.edu.cn

**Keywords:** *Monascus*, pigment, metabolomics, weighted gene co-expression network analysis, tricarboxylic acid cycle

## Abstract

*Monascus* azaphilones pigments (MonAzPs) produced by microbial fermentation are widely used as food chemicals for coloring and supplying beneficial biological attributes. In this study, a fermentation perturbation strategy was implemented by separately adding different amino acids, and detecting the intracellular metabolome via UHPLC-Q-Orbitrap HRMS. With the aid of weighted gene co-expression network analysis, two metabolic intermediates, fumarate and malate, involved in the tricarboxylic acid cycle, were identified as the hub metabolites. Moreover, exogenous addition of fumarate or malate significantly promoted red pigment production, and reduced orange/yellow pigment production. The importance of the tricarboxylic acid cycle was further emphasized by detecting intracellular levels of ATP, NAD(P)H, and expression of oxidoreductase-coding genes located in the MonAzPs synthetic gene cluster, suggesting a considerable effect of the energy supply on MonAzPs synthesis. Collectively, metabolomics is a powerful approach to position the crucial metabolic regulatory factors, and facilitate the development of engineering strategies for targeted regulation, lower trial-and-error cost, and advance safe and controllable processes for fermented food chemistry industries.

## 1. Introduction

**Figure 1:**
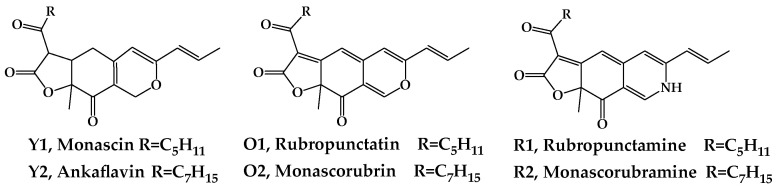
The major six molecular structures of MonAzPs.

*Monascus* spp. are important edible food microbial resources in Asian countries (China, Korea, Japan, Thailand, Indonesia, and the Philippines), producing a variety of secondary metabolites, such as pigments, alcohols, antibiotics, antihypertensives, and taste compounds, that are extensively used to improve the flavor and quality of foods [1,2,3]. These metabolites have a wide range of healthy physiological attributes, including cardiovascular disease prevention, anti-inflammation, gastrointestinal, and digestive system improvement, and cancer prevention [4,5]. The natural edible food *Monascus* azaphilones pigments (MonAzPs) produced by *M. purpureus* are widely utilized as food coloring agents due to their high coloring performance, ease of manufacture on low-cost substrates, as well as their possessing a variety of healthy attributes [6,7,8]. Among the more than 100 of the known MonAzPs, 6 major MonAzPs are monascin and ankaflavin (yellow, Y1 and Y2), rubropunctatin and monascorubrin (orange, O1 and O2), and rubropuctamine and monascorubramine (red, R1 and R2) (Figure 1), whose physicochemical and nutritive properties have been well studied [9].

Due to the importance of MonAzPs in food, health care, and medicine industries, more study has concentrated on the production of MonAzPs and the biochemical synthetic pathway, in order to improve the manufacturing process. MonAzPs are generally generated by the polyketide biosynthesis pathway, the fatty acid synthesis pathway, and the post-synthetic modification pathway [5]. The diversity of MonAzPs molecular genetics originates from the *Monascus* unitary trunk pathway, which directs intermediates to the typical food yellow and orange MonAzPs and includes a number of shunt pathways branching off from the trunk pathway [10]. Many factors influence the formation and differentiation of MonAzPs, including: (1) gene cluster expression [11,12,13,14]; (2) the availability of universal precursors, such as acetyl-CoA and malonyl-CoA [15]; (3) the supply of cofactor NAD(P)H and ATP [16]; and (4) enzyme catalysis selectivity. Furthermore, uncatalyzed *O*-to-*N* substitution reactions with accessible amines in the cell or culture broth can form a wide range of red MonAzPs with a *g*-vinylogous pyridine core, thus complicating pigment species and yield regulation [10,17]. Therefore, external conditions, such as carbon source [18], nitrogen source [19,20,21], surfactant [22,23], dissolved oxygen [24], pH [25], temperature [26], redox potential [27], light [28], and low frequency magnetic field [29], have considerable impacts on food MonAzPs synthesis by interfering with intracellular metabolism.

Taken together, the existence of multifactor regulation leads to the fact that quite a number of exogenous and endogenous factors could give rise to the variation of food MonAzPs production. Such multi-dimensional metabolic regulations force *Monascus* to adapt to different cultivation conditions for growth, but consequently leave *Monascus* fermentation to be hardly controlled for the specific MonAzPs components or their combinations. Similar cases are also involved in many other complex biosynthetic pathways, especially in edible fungi that often detail relatively simple carbon skeletons from polyketide biosynthesis to produce a large variety of secondary metabolites, which define the final characteristics of the products, such as nutritional quality and safety.

Understanding the complexity of such a system like MonAzPs regulation, and identifying and associating biochemical changes with the desired product quality characteristic, largely depend on the experimental and analytical techniques feathered with a powerful ability related to systematic analysis [30]. The metabolomics approach is critical for addressing this issue because it uniquely provides diagnostic patterns via fingerprinting, absolute quantitation of targeted metabolites, relative quantitation of large portions of the metabolome using metabolite profiling, and tracing of individual metabolite biochemical fate through a metabolic system. Similarly, advances in bioinformatics identification algorithms over recent decades have enabled quantitative measurements of relative or absolute metabolite amounts in cells, foods, and intestinal contents in a high-throughput manner, which has significantly accelerated metabolomics research into the functions and dynamics of complex biological systems [31].

Omics analysis is highly dependent on diverse computational approaches, with network-focused strategies providing a more full view of biological responses than individual gene/protein/metabolite-focused strategies [32]. Weighted gene co-expression network analysis (WGCNA) has been widely used to describe correlation relationships between clusters of highly correlated genes/metabolites or modules and external conditions or sample traits, owing to its systems-level insight and high sensitivity to low abundance or small changes without any information loss compared to unweighted networks [33,34,35].

Therefore, we present a hypothesis in this study that UHPLC-Q-Orbitrap HRMS and WGCNA-based metabolomics could reveal the associations of phenotypical variation in food MonAzPs fermentation by *M. purpureus* with intracellular metabolic intermediates, and provide robust fermentation regulation strategies of such significant food chemicals with healthy physiological activities. To the best of our knowledge, this is the first report of WGCNA-based metabolomics analysis of MonAzPs fermentation by *M. purpureus* to position the crucial metabolic regulatory factors, and facilitate the development of engineering strategies for targeted regulation for MonAzPs fermentation.

## 2. Materials and Methods

### 2.1. Strains and Media

Strain. *M. purpureus* M7 [20] was originally isolated from the red rice sample (Hong Qu) in Gutian County, Ningde, China, and deposited in our laboratory.

Media. Potato dextrose agar (PDA) solid medium for producing spores (g/L): potato extract 4.0, dextrose 20.0, agar 15.0. Mature spores of *M. purpureus* M7 were harvested and stored in a 20% (*v*/*v*) glycerin aqueous solution at −80 °C until needed. Seed medium (g/L): rice flour 30.0, NaNO_3_ 3.0, KH_2_PO_4_ 2.5, MgSO_4_·7H_2_O 1.0, and pH 6.0. Fermentation media (g/L): glucose 50.0, (NH_4_)_2_SO_4_ 5.0, KH_2_PO_4_ 4.0, MgSO_4_·7H_2_O 0.5, MnSO_4_·H_2_O 0.03, ZnSO_4_·7H_2_O 0.01 and FeSO_4_·7H_2_O 0.01, and pH 6.8.

Cultivation methods. Spore suspension (1 mL) was transferred to seed medium (40 mL) in a 250 mL Erlenmeyer flask incubated at 30 °C and 180 rpm, for 48 h in a shaking incubator (ZQWY-200N, Shanghai Zhichu Instrument Co., Ltd., Shanghai, China). Seed culture (4 mL) was added to the fermentation medium (40 mL) in a 250 mL Erlenmeyer flask and cultured in the shaking incubator (180 rpm, 30 °C) for a 4-day fermentation.

Fermentation perturbation experiments were conducted by adding 17 kinds of amino acids into the fermentation media, respectively, with a final concentration of 0.2 g/L, while the group without any addition was used as the control. These amino acids were glutamic acid, aspartic acid, lysine, arginine, histidine, leucine, isoleucine, alanine, phenylalanine, threonine, serine, proline, tyrosine, methionine, valine, glycine, and tryptophan. The fermentation experiment was repeated at least three times.

### 2.2. Analytical Methods

#### 2.2.1. Dry Cell Weight (DCW) and Glucose

Fermentation broth (5 mL) was centrifuged at 2000× *g* for 15 min (TDZ5-WS, Xiangyi Centrifuge Instrument Co., Ltd., Changsha, China). The obtained mycelium pellet was washed once with deionized water, then dried to a constant weight at 60 °C. DCW was recorded by electronic precision balance. At the same time, the supernatant was filtered through a 0.22 µm filter for determining the glucose concentration. The glucose detection system was UltiMate 3000 HPLC (Thermo Fisher Scientific Inc., Waltham, MA, USA) equipped with a Bio-rad Aminex HPX-87H column and a refractive index detector. The mobile phase was 5 mM sulfuric acid aqueous solution with a flow rate of 0.6 mL/min at 60 °C.

#### 2.2.2. MonAzPs Production

The fermentation medium (2 mL) was mixed with aqueous ethanol (8 mL, 70%, *v*/*v*), maintained at 80 °C for 1 h, then ultrasonicated (KQ8200B, Kunshan Ultrasonic Instrument Co., Ltd., Shanghai, China) with a maximum power for 30 min. After centrifugation at 2000× *g* for 15 min, the supernatant was filtered through a 0.22 μm filter for MonAzPs determination by HPLC (1200 Infinity, Agilent Technologies, Santa Clara, CA, USA), equipped with a ZORBAX SB-C18 column (Agilent) and a DAD detector set to 410 nm. The mobile phase was 0.1% (*v*/*v*) formic acid–water solution and acetonitrile at a ratio of 65:35 (*v*/*v*) at 1.0 mL/min, 25 °C. The concentrations of monascin, ankaflavin, rubropunctatin, monascorubrin, rubropunctamine, and monascorubramine were calculated by standard curves of pigment standards.

#### 2.2.3. Metabolomics Experiment

Sample preparation. The mycelia of 5 mL fermentation broth were collected and washed in precooled phosphate-buffered saline (PBS) (KH_2_PO_4_ (0.24 g/L), Na_2_HPO_4_ (1.44 g/L), NaCl (8 g/L), KCl (0.2 g/L), dissolved in deionized water, pH 7.2). Wet mycelia (1.0 g) were treated with methanol/acetonitrile/H_2_O (1.5 mL; 40:40:20 *v*/*v*/*v*) at −20 °C for 60 min to extract intracellular metabolites. After freezing and thawing in liquid nitrogen at least five times, extract was centrifuged at 5000× *g* at 4 °C for 10 min and supernatant was retained to determine metabolite profiles.

Data acquisition and processing. Metabolite determination was carried out using ultra-high-performance liquid chromatography-Q exactive hybrid quadrupole orbitrap high-resolution accurate mass spectrometry (UHPLC-Q-Orbitrap HRMS), as described previously with negative ion mode [36] in Shanghai Applied Protein Technology Co., Ltd. Xcalibur 4.0 software (Thermo Fisher, Waltham, MA, USA) was used for data acquisition and processing. Identification of metabolites was achieved by high-resolution mass and retention-time matching to authentic standards. Metabolite abundances were normalized to wet mycelium weight. The metabolomics experiment was biologically repeated twice.

#### 2.2.4. Construction of WGCNA Network

WGCNA was built based on the metabolomics data, followed by the online tutorial [37] with R 4.1.0 [32]. In brief, a similarity matrix was created by calculating Spearman rank correlation coefficients between metabolite profiles across all samples. Based on the free-scale topology criterion, the matrix was transformed into an adjacency matrix raised to an exponent (soft threshold). Through hierarchical clustering, the dissimilarity matrix (1-TOM, TOM refers to Topological Overlap Matrix) was employed to construct modules clustered by metabolites with highly comparable correlation relationships. Each metabolite module was allocated a standard RGB color by default. The correlation between the module eigen-metabolite and the physicochemical characteristics, such as DCW, glucose, and MonAzPs production was used to estimate module–trait associations. Metabolites with significant module–trait associations (*p*-value < 0.05) within modules were assigned for the identification of hub metabolites that have high intramodular connectivity, high module membership (MM), and metabolite significance (MS).

#### 2.2.5. Fermentation Regulation by Supplementing Hub Metabolites

To validate the effects of biomarkers proposed by WGCNA on the production of food MonAzPs, the three compounds were, respectively, added into the control media with a final concentration of 0.05 g/L, combined with the quantitative determination of the six pigments and mycelia biomass.

#### 2.2.6. qRT-PCR Analysis of the Expression of Oxidoreductase-Coding Genes Located in MonAzPs Synthetic Gene Cluster

Total RNA was extracted using an RNA prep pure Cell/Bacteria kit (CWBIOTECH Inc., Beijing, China), following the manufacturer’s instructions. cDNA was obtained by reverse transcription with total RNA as the template, using a PrimeScript™ RT reagent kit (CWBIOTECH, Beijing, China). Genome of *M. purpureus* YY-1 [38] was used as the template to design primer sequences (Table 1), with the gene *Actin* (C5.619) as the internal reference. qRT-PCR was carried out on a 7500 Real-Time PCR System (Applied Biosystems Inc., Foster, CA, USA), using Q-PCR was performed by using 2 × RealStar Green Fast Mixture (Genstar, Beijing, China), following the 2^−ΔΔCt^ method [39]. The PCR temperature program was: 50 °C, 2 min; 95 °C, 10 min; 60 °C, 1 min; 95 °C, 15 s; 60 °C, 1 min; 95 °C, 30 s; 60 °C, 15 s, maximum cycle number 35. The qRT-PCR analysis was repeated at least three times.

#### 2.2.7. Determination of Intracellular Levels of ATP, NAD(P)^+^ and NAD(P)H

The levels of intracellular ATP, NADH, and NADPH were determined using ATP Assay Kit, NAD^+^/NADH, and NADP^+^/NADPH Assay Kits (Suzhou Grace Biotechnology Co., Ltd., Suzhou, China) according to manufacturers’ instructions. Three biological repeats were conducted for the above analysis.

If not specified otherwise, Student’s t test for independent samples was used for statistical analysis by R 4.1.0.

## 3. Results

Growth perturbation is a common strategy to investigate cellular systems, in which employing the growth conditions, such as pH, temperature, nutrients, and gene manipulation, gives rise to the stimulation on microorganisms to generate metabolic variation and adaptive adjustment to reach a new steady state consequently, at the cost of phenotypic changes on growth rate, sugar consumption and the product yield. Due to its strong adaptability, *Monascus* spp. incorporates different forms of nitrogen sources into the metabolic systems through hydrolysis, transport and transformation, and produces a series of target food products, accompanied by the adjustment of intracellular metabolism. Monitoring phenotypic and intracellular characteristics are the basis of understanding the adaptive metabolic regulation. Therefore, in this study, a physiological perturbation experiment was conducted by exogenously adding small amount of amino acids (0.2 g/L) into the fermentation media, combined with the determination of food MonAzPs production profiles, biomass, glucose consumption, as well as the intracellular metabolome.

### 3.1. Fermentation Characterization and the Variations of MonAzPs Production Perturbation by M. purpureus

**Figure 2:**
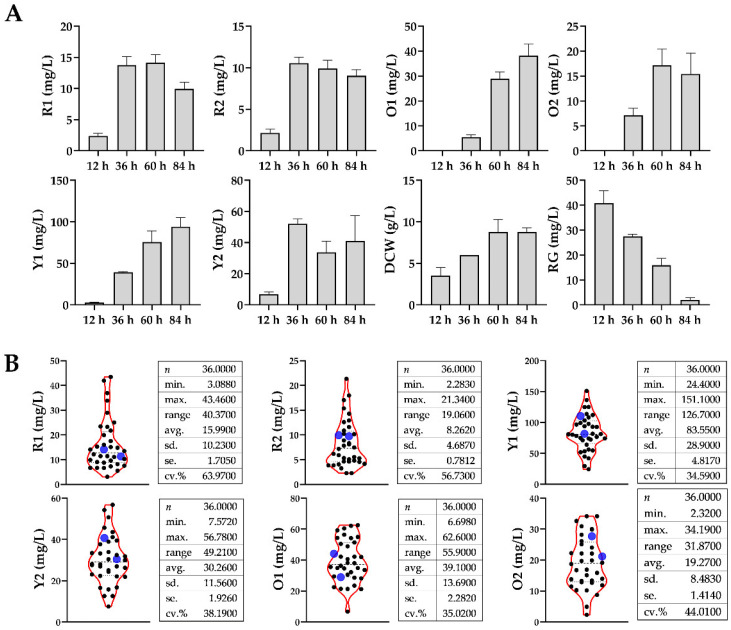
(**A**) Time-coursed characterization of *Monascus* pigment production, biomass, and glucose consumption in the control group. The error bar represents the standard deviation of three independent experimental data. RG, residual glucose in the fermentation broth. (**B**) Distribution of pigment production at 60 h under metabolic perturbations with different amino acids. n, number of samples; min., minimal value; max., maximum value; range, data variation range (max.–min.); avg., average value; sd., standard deviation; se., standard error; cv., coefficient of variance.

As shown in Figure 2A, using (NH_4_)_2_SO_4_ as the basal nitrogen source (control group), phenotypic characteristics of *Monascus* fermentation at different phases were recorded. Based on profile of mycelial biomass, *M. purpureus* growth obviously fell into two phases, i.e., ascending phase (12~60 h) and declining phase (60~84 h). It was found that the production of R1, R2, and O2 reached the peaks in the middle period of fermentation, whereas O1 and Y1 increased continuously during the whole period. Interestingly, the production of Y2 reached the maximum at 36 h, decreased at 60 h, then slightly rebounded at 84 h.

It could be seen that fermentation at 60 h appeared to be a key observation point to distinguish different MonAzPs production profiles, as well as the watershed between the ascending phase and declining phase. Hence, it was used as the sampling point for detecting the metabolic perturbations by adding different amino acids. Each fermentation broth sample was divided into two parts, one for the extraction and detection of metabolome, and the other for the detection of pigment production.

Violin plots of Figure 2B describe the distribution of MonAzPs production, where blue scatters represented the values of control groups. As depicted, exogenous addition of different amino acids caused extensive phenotypic differences in that the distribution of MonAzPs production was spindle-shaped, and the data were randomly distributed above and below the control group. Moreover, the coefficients of variation (CV, the ratio of standard deviation to average) in each dataset varied from 0.3459 to 0.6397, indicating that the phenotypic variation was moderate and external interference did not yet lead to a serious metabolic collapse of *Monascus*.

### 3.2. Metabolomics Analysis Coupled with WGCNA Modelling

The UHPLC-Q-Orbitrap HRMS used in this study allows for complete scan in a precise and fast way, providing reliable and substantive details about molecular weight and structure. According to the metabolomics analytical procedure, a total of 68 metabolites, mainly including phosphate compounds, amino acids, and low molecular weight carboxylic acids (Table S1) were identified by the UHPLC-Q-Orbitrap HRMS system and verified by matching mass and retention time to authentic standards. To search for the associations between phenotypes and metabolomes, a systematic biological method, WGCNA, was employed to obtain a network-focused picture of cellular responses.

**Figure 3:**
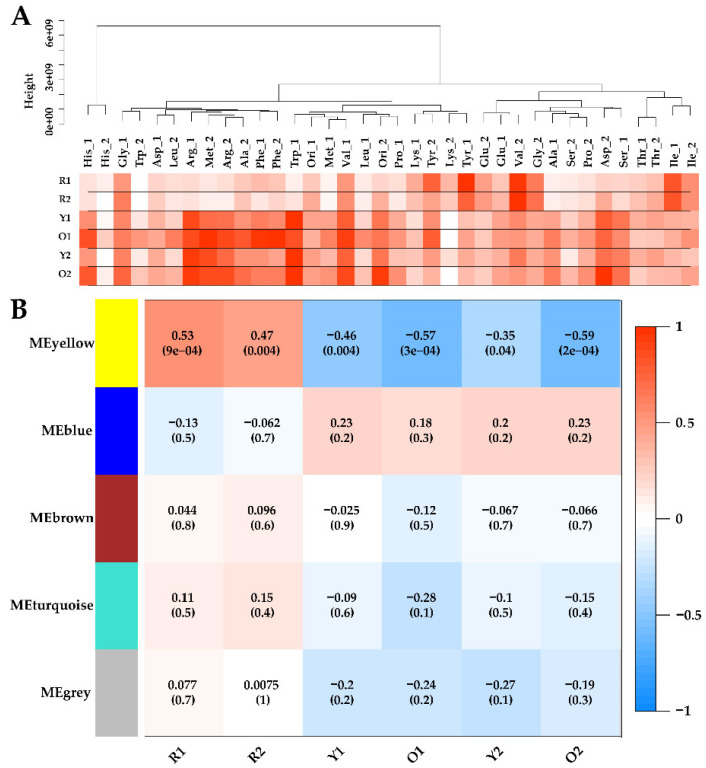
(**A**) Clustering dendrogram of metabolomics samples based on their Euclidean distance, linked to the heatmap of pigment production. The levels of pigment production are proportional to color density. (**B**) Module–trait associations. Each row corresponds to a module eigengene, column to a trait. Each cell contains the corresponding correlation coefficient and *p*-value.

According to the recommended procedure, metabolome samples were clustered and connected with the phenotypic data by WGCNA as shown in Figure 3A. Obviously, adding tyrosine or valine significantly promoted the production of red MonAzPs (R1 and R2), compared to the other amino acids. However, the production of Y1, O1, Y2, and O2, were highly variable in all the samples. Such extensive phenotypic diversity indicated the self-regulation and homeostasis of *Monascus* metabolism, and it was suited for establishing the quantitative relationship between intracellular and extracellular properties using correlation analysis.

For the construction of WGCNA, the soft-threshold was set at 7 for the criterion of free-scale topology, and 68 metabolites were grouped into four modules (module MEgrey contained unrelated metabolites and was excluded in WGCNA) [40]. Module–trait relationships were determined using correlations between the module eigen-metabolites and MonAzPs production, which enabled the identification of co-occurrence of metabolite sets with significant correlations to physiological characteristics. Consequently, one module–trait relationship (MEyellow in Figure 3B) was identified as *p*-value < 0.05. Interestingly, the MEyellow module was positively correlated with R1 (*r* = 0.53, *p* = 9 × 10^−5^), and R2 (*r* = 0.47, *p* = 4 × 10^−3^), and was significantly negatively associated with Y1 (*r* = −0.46, *p* = 4 × 10^−3^), O1 (*r* = −0.57, *p* = 3 × 10^−4^), Y2 (*r* = −0.35, *p* = 4 × 10^−2^), and O2 (*r* = −0.59, *p* = 2 × 10^−4^).

**Figure 4:**
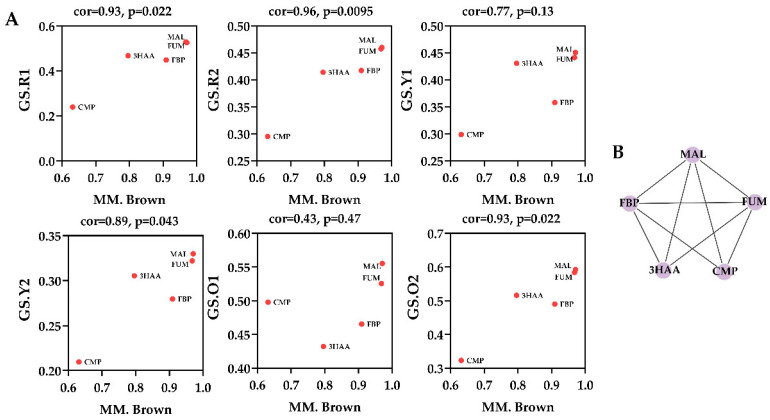
Identification of hub metabolites. (**A**) Scatterplot of MS vs. MM in the brown module. (**B**) Intramodular connections within the brown module. FUM, fumarate; MAL, malate; CMP, cytidine monophosphate; FBP, fructose 1,6-bisphosphate; 3HAA, 3-hydroxyanthranilic acid.

Within WGCNA networks, highly connected metabolites, also called hub metabolites, play an important role in understanding the biological mechanism of response under stresses/conditions. Hub metabolites are usually featured with the greatest metabolite significance (MS), module membership (MM), and the connectivity with other node metabolites, in which MS is defined as a *p*-value of each metabolite in the linear regression between metabolite abundance and the phenotypes, and MM is a measure of intra-modular connectivity. For the MEyellow module, a scatterplot of MS vs. MM is presented in Figure 4A. The MEyellow module contained five metabolites, i.e., fumarate, malate, cytidine monophosphate, fructose 1,6-bisphosphate, and 3-hydroxyanthranilic acid, of which fumarate and malate were identified as the hub metabolites since they were characterized with the highest MM and MS values, as well as the biggest connectives in Figure 4B.

As a conclusion of WGCNA analysis, two metabolites, fumarate and malate, were identified as the hub metabolites that had extensive and close relationships with other metabolites within the MEyellow module. The previous analysis on module–trait associations (Figure 3B) showed that MEyellow module was related to MonAzPs production. Therefore, it is reasonable to speculate that these two metabolites, as hubs of the module, have an effect on MonAzPs production. Considering the biological importance of fumarate and malate involving in reduced tricarboxylic acid cycle (reTCA), a metabolic perturbation experiment aimed at directed regulation of reTCA was proposed to further investigate the significance of these two metabolites. Additionally, oxaloacetate, due to the tight relationship with malate, and the well-known regulatory effect on the TCA cycle, was also investigated.

### 3.3. Fermentation Regulation by Supplementing Hub Metabolites

**Figure 5:**
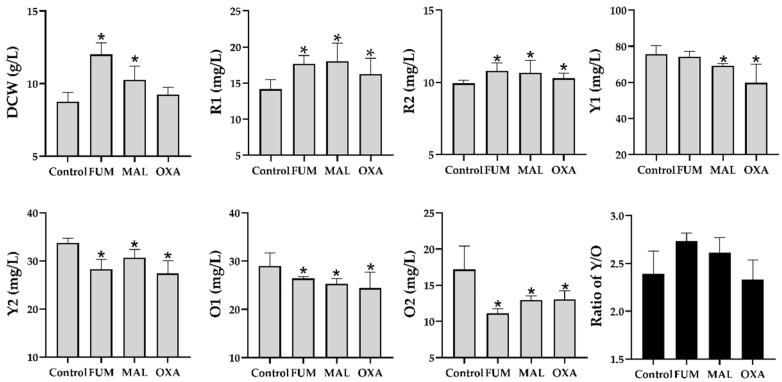
Effects of supplementing biomarkers on the MonAzPs production and mycelia biomass. Each experiment was repeated at least three times. Data were shown as mean ± error bars. Significance of the difference between treated and the control group was analyzed by Student’s t-test. * *p* < 0.05. MAL, malate; FUM, fumarate; OXA, oxoacetate. Ratio of Y/O was calculated by formula of (Y1 + Y2)/(O1 + O2).

To validate the physiological effects of fumarate, malate, and oxaloacetate on the production of MonAzPs, the three compounds were, respectively, added into the control media. As depicted in Figure 5, yields of two red MonAzPs, R1 and R2, and in malate- and fumarate-treated groups, were higher than those of the control group at 60 h (*p* < 0.05, not significant in oxoacetate-treated group with R2). The other MonAzPs’ productions, i.e., Y1, Y2, O1, and O2, collectively, were decreased when adding fumarate, malate, or oxaloacetate (*p* < 0.05 except in Y1 of fumarate group). These findings suggested the effectiveness of adding hub metabolites for regulating the MonAzPs’ production.

### 3.4. Associations between TCA Cycle and MonAzPs Synthetic Pathway

The TCA cycle is the common oxidative pathway for carbohydrates, fats, and amino acids, which is the most important metabolic pathway for the energy supply to aerobic organisms. The cycle oxidizes pyruvate to CO_2_ and H_2_O, with the concomitant production of energy (ATP) from NADH and FADH_2_ [41] via electron transport chain. In addition, the cycle can produce NADPH through the activity of isozymes that reside either in the mitochondria (isocitrate dehydrogenase) or in the cytosol [42]. As an important measure of intracellular redox state, an elevated intracellular NADH:NAD^+^ ratio, the so-called “reductive stress”, or a lower ratio, “oxidative stress”, is crucial for numerous fundamental cellular processes, including energy metabolism, calcium homeostasis, cell death, and proliferation [43]. The cofactor-dependent redox reactions, meanwhile, are vital for the MonAzPs biosynthesis, including the formation of MonAzPs’ chromophore structures mediated by MpigC (C-11-ketoreductase), MpigN (monooxygenase) and MpigE (NAD(P)H-dependent oxidoreductase), and the conversion of yellow/orange MonAPs with the aid of MpigF (oxidoreductase) and MpigH (enoyl reductase). Therefore, we determined the NAD(P)H:NAD(P)^+^ ratios compared to the control group after supplementing fumarate, malate, and oxaloacetate.

**Figure 6:**
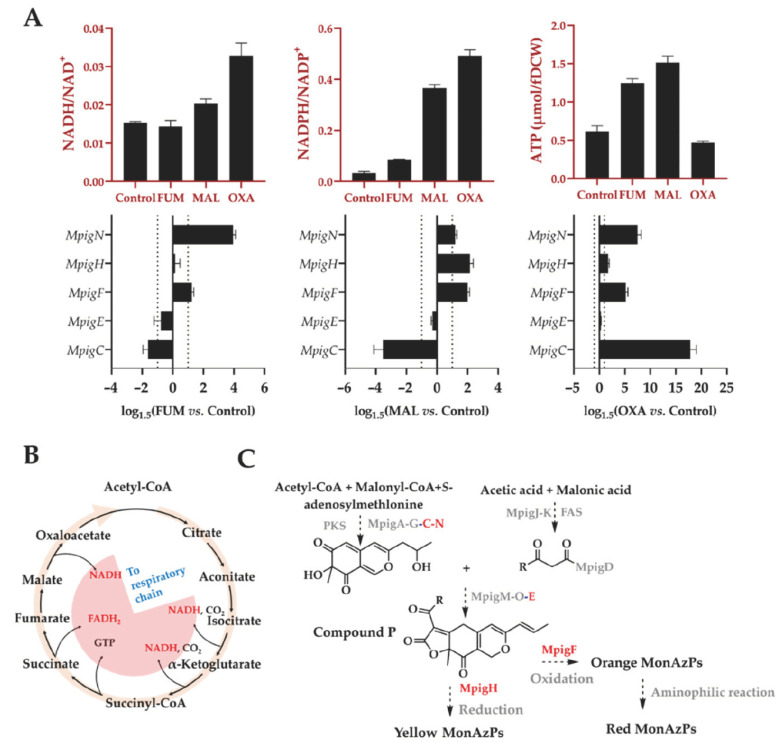
(**A**) Changes of ATP, reductive equivalents (NADH, NADPH), and the expressions of dehydrogenase genes involving with MonAzPs biosynthesis after supplementing fumarate, malate, and oxoacetate, respectively; (**B**) TCA cycle; and (**C**) MonAzPs synthetic pathway. MpigA, nonreducing polyketone synthase; MpigB, transcription factor; MpigC, C-11-ketoreductase; MpigD, 4-O-Acyltransferase; MpigE, NAD(P)H-dependent oxidoreductase; MpigF, oxidoreductase; MpigG, serine hydrolase; MpigH, enoyl reductase; MpigJ, FAS subunit α; MpigK, FAS subunit β; MpigM, O-acetyltransferase; MpigN, monooxygenase; MpigO, deacetylase; MpigI, transcription factor; MpigL, ankyrin repeat protein; MpigP, major facilitator superfamily multidrug transporter.

As shown in Figure 6, the NADPH: NADP^+^ ratios in all the treated groups were enhanced, particularly in malate and oxaloacetate groups along with the higher NADH: NAD^+^ ratios (no significant difference of NADH/NAD^+^ in fumarate group vs. control). These findings demonstrated the feasibility of supplementing these compounds on regulating the intracellular redox state through TCA cycle. As a consequence, the elevated NAD(P)H:NAD(P)^+^ ratio promoted the formation of “reductive stress”, thus driving NADH-mediated reduction reactions, especially for the conversion of orange MonAzPs (O1 and O2) into the yellow MonAzPs Y1 and Y2, which was supported by the increased ratio of yellow pigments to the orange pigments (Y/O) in the treated groups (Figure 5).

Given that in micro-organisms under aerobic conditions, most ATP production comes from oxidative phosphorylation pathway that is tightly associated with the NAD(P)H availability, and the electron transfer chain activity [44], the intracellular ATP concentration was determined in this study. As shown in Figure 6, ATP concentration supplemented with fumarate or malate was significantly higher than that of control, consistent with the changing profile of intracellular NAD(P)H. Interestingly, supplementing oxoacetate depressed the generation of ATP, whereas it significantly promoted the accumulation of NAD(P)H, suggesting a low conversion efficiency of oxidative phosphorylation chain, which might be related to the lower Y/O ratio in oxoacetate group (Figure 5).

In addition to the effect of intracellular energy metabolism, the expressions of *MpigC*, *E*, *F*, *H*, and *N* located in the MonAzPs synthetic gene cluster that mediates redox reaction during pigment synthesis, were determined by qRT-PCR. Among them, three oxidoreductases, MpigC, MpigN, and MpigE, are involved with the biosynthesis of the MonAzPs’ chromophore core, whereas the other two oxidoreductases, MpigF and MpigH, are associated with the synthesis and differentiation of orange and yellow MonAzPs, respectively. As depicted in Figure 6, compared to the control: (1) *MpigC* was down-regulated in fumarate and malate groups, but up-regulated in oxaloacetate group; (2) *MpigN* and *MpigF* were up-regulated in all three experimental groups; (3) the expression change of *MpigE* was not significant under all three conditions; and (4) *MpigH* was up-regulated in malate and oxaloacetate groups. Upregulation of *MpigF* and *MpigH*, was expected to be closely related with the production improvement of yellow or orange MonAzPs, but unexpectedly, it led to the opposite results. These findings suggested a distant association between the pigment production and the gene cluster expressions, and the gene cluster expression was not the limiting factors during the production of MonAzPs.

## 4. Discussion

Natural edible food MonAzPs and other secondary nitrogenous metabolites appear insignificant to the growth of *Monascus*, and their productions often suffer a major setback when adapting to environmental changes. In this study, adding single amino acid of a small amount generated extensive metabolic perturbations, resulting in the alterations of phenotypic characteristics of *M. purpureus* M7. However, the stability of production and product quality of MonAzPs is exclusively critical for *Monascus* fermentation in industry. Until now, numerous factors, including: pH; temperature; carbohydrates; light; and especially nitrogen sources, such as peptone, yeast powder, ammonium chloride, and ammonium nitrate; have been proved to lead to different effects on MonAzPs production [21], which undoubtedly complicates the investigation of *Monascus* fermentation. Hence, a simplified and efficient model, such as metabolic perturbation, is necessary to dissect the metabolic mechanism of MonAzPs biosynthesis and regulation, and aid to establish a high-efficient fermentation regulation strategy.

*Monascus* spp. naturally produces several extracellular proteases and peptidases mediating the hydrolysis of proteins into amino acids as the nutrition for the growth and development [45]. Amino acids either directly participate in the protein synthesis, or decompose into intermediates of glycolysis, TCA, and pentose phosphate pathways, mainly involved with the primary metabolisms. The abundance of metabolites and their fluxes are not only the participants of intracellular biochemical reactions generating intermediates and macromolecules, but also the end products of metabolism. It is considered to be closely associated with the fermentation phenotypes (such as target products and biomass) [46,47]. In this study, the metabolomics approach was used to profile intracellular metabolism changes in response to various amino acids through qualitative and quantitative analysis. By clustering analysis, we can clearly distinguish the similarities and differences of amino acids’ impacts on intracellular metabolic changes. However, this study aims not only to explain how different amino acids produce an effect on the intracellular metabolome, but also to explore the regulatory mechanism of MonAzPs’ biosynthesis, so as to provide holistic insights into the targeted regulation and control of their production. To emphasize the intrinsic relevance among metabolites, a WGCNA method derived from the gene co-expression analysis [31,32], was adopted to identify the biomarkers that have a significant impact on MonAzPs’ production. By clustering the metabolites with similar change profiles in the metabolome and evaluating their relationships with the extracellular phenotype, the so-called WGCNA module was recognized and associated with MonAzPs’ production. Furthermore, fumarate and malate were identified as the hub metabolites within the key WGCNA module. The intracellular abundance of these two metabolites may have a significant impact on MonAzPs’ production, providing clues for regulating pigment biosynthesis.

Fumarate and malate, as important intermediates of the TCA cycle, play an important role in driving TCA metabolic flow and promoting TCA derived energy metabolism [41,48]. Based on the above speculation, there followed a supplementing strategy being put forward, and we observed the significant changes in MonAzPs’ production, intracellular ATP, and NAD(P)H. Collectively, adding these biomarkers, led to a higher level of red MonAzPs, whereas lowering the yellow and orange MonAzPs. With high levels of reducing equivalents, more ATP were synthesized through oxidative respiratory chain, but interestingly, adding oxaloacetate depressed the intracellular ATP level, which might be due to its shunt flux between TCA and EMP. Additionally, by regulating the TCA cycle, we found that gene expression encoding oxidoreductases in *Monascus* synthesistic gene cluster changed greatly, but it made little contribution to explaining the production change, possibly because gene cluster expression was not the main limiting factor of pigment synthesis under the conditions used in this study, as described previously [21].

Despite the fact that MonAzPs are biosynthesized as secondary metabolites in the middle and late stages of *Monasucs* fermentation, their production is inextricably linked to primary metabolism. Previously, we discovered that the pentose phosphate pathway influenced the synthesis of yellow pigments [20], and intracellular amino acids influenced the synthesis of red MonAzPs [21], while orange MonAzPs, as the precursors of yellow and red MonAzPs, were subject to more complex regulation. Because of the diversity of MonAzPs molecules (over a hundred have been found to date), determining that their interaction in biosynthesis is crucial for targeted regulation of specific pigment production. The genetic basis of *Monascus* pigment diversity has been largely clarified by Chen et al. [10] through gene knock-out and overexpression within pigment synthetic gene cluster, but the production of MonAzPs is dynamic, being constantly influenced over time by the combined effects from chassis micro-organisms (different strains), precursor compounds (such as acetyl-CoA and malonyl-CoA) [15], energy supply (NAD(P)H and ATP) [16], expression of gene clusters and enzyme activities. Therefore, to learn more about the regulatory mechanism of *Monascus* pigment production, transcriptome, proteome, and metabolome investigations are necessary.

## 5. Conclusions

Collectively, adding amino acids generated a perturbation on MonAzPs’ production, confirming the variation of pigment biosynthesis when changing the medium components. Such a complicated situation will force us to redesign the culture conditions and medium components to maintain the pigment production level. Fortunately, in this study, UHPLC-Q-Orbitrap HRMS-based metabolomics analysis pointed out that different amino acids exerted the most significant effect in the TCA cycle via fumarate and malate. Based on this hypothesis, the supplementation of biomarkers, and the determination of intracellular levels of ATP, NAD(P)H, and crucial gene expressions confirmed that two metabolic intermediates, fumarate and malate, impacted the pigment biosynthesis. Subsequently, associations between TCA cycle and the pigment biosynthesis pathways were comprehensively investigated. Due to the close relationship of TCA cycle with dissolved oxygen and respiration chain, more regulation strategies of *Monascus* fermentation could be put forward in the future. More importantly, targeted exogenous addition strategies reduce the trial-and-error cost, and are safe and controllable for fermented food industries. The result is expected to provide theoretical framework and support for the research of food MonAzPs synthesis and engineering strategies development for targeted regulation, and subsequent quality and safety supervision for fermented food chemistry industries.

## Figures and Tables

**Table 1:** Primers for qRT-PCR in this study.

*Gene*	Sense Primer	Anti-Sense Primer
*Actin*	ATCCCGTCCTCCTGACTGAA	CCTCGTAGATGGGAACGACG
*MpigC*	GCGGCGCATCTTCTCAACC	TCGTCCTCCAAGCCAGTCCC
*MpigE*	CCCCTGTCTGCGTGCGTAT	AGTAGCGGTGCCGGTGATG
*MpigF*	AGCCGCAAACTCGTCCTCG	CCTCGGTCAACGCAGTCTCG
*MpigH*	ATTTGGTCCCACGGGCTTCT	TCCAACGCCCTCGTGTCCT
*MpigN*	GCATCATCACCGCACCA	GCATCACGACCAGTAGGC

## Data Availability

The datasets used and/or analyzed during the current study are available from the corresponding author on reasonable request.

## Supplementary Materials

The following are available online at https://www.mdpi.com/article/10.3390/foods11142168/s1. Table S1: Metabolite abundance in each sample.
